# Atraumatic Maxillary Canine Root Repositioning as an Alternative to Orthodontic Forced Eruption: A Case Report

**DOI:** 10.1002/ccr3.72016

**Published:** 2026-02-10

**Authors:** Shamsedin Heydari, Mohsen Aminsobhani, Foozie Zahedi

**Affiliations:** ^1^ Department of Endodontics, School of Dentistry Tehran University of Medical Sciences Tehran Iran; ^2^ Department of Oral and Maxillofacial Radiology, School of Dentistry, Dental Implants Research Center Hamadan University of Medical Sciences Hamadan Iran

**Keywords:** atraumatic extraction, dental implant, minimally invasive surgical procedures, tooth extraction

## Abstract

Preservation of natural teeth in the anterior region is often preferred over replacement with dental implants due to functional and esthetic considerations. Although orthodontic extrusion preserves soft tissue architecture, it may not be suitable in all cases. Atraumatic extrusion using minimally invasive extraction systems allows controlled coronal repositioning of teeth while preserving periodontal structures. This case report presents the clinical application of atraumatic root repositioning of a maxillary canine as an alternative to orthodontic forced eruption. The findings suggest that this technique can facilitate restoration, reduce treatment morbidity, and promote favorable biological healing in selected cases.

## Key Clinical Message

Atraumatic surgical extrusion using a winch‐based extraction system can be a minimally invasive and accessible alternative to orthodontic forced eruption in severely compromised anterior teeth. This technique facilitates restoration by preserving periodontal structures, improving isolation, and enabling favorable placement of restorative margins.

## 1 Introduction

Preservation of natural teeth through root retention and coronal reconstruction offers several benefits over tooth replacement options like implants, including maintaining proprioception, conserving alveolar bone, minimizing surgical needs, and reducing costs [1].

Despite advances in implantology, its application in the anterior maxilla remains limited due to challenges such as thin buccal bone (< 1 mm in 90% of cases) and proximity to neurovascular structures, which contribute to post‐extraction bone loss and complicate implant placement [2, 3, 4, 5]. Thus, retaining natural teeth, particularly in the esthetic zone, is often a more favorable option [6]. Teeth with significant coronal damage—such as cervical caries, subgingival fractures, or extensive structural loss—require crown lengthening prior to restoration [7, 8, 9].

Traditional crown lengthening through osteotomy may compromise adjacent bone and soft tissue, leading to gingival recession and hypersensitivity [10, 11]. As a less invasive alternative, Ingber introduced forced eruption in 1974 [12], later refined with supracrestal fibrotomy to prevent periodontal tissue rebound [13]. This technique repositions the tooth supragingivally to create a ferrule and reestablish biologic width [7].

Orthodontic extrusion, while preserving soft tissue and esthetics [12, 14], is time‐consuming and unsuitable for teeth with poor root morphology, short root length, or compromised periodontal status [15].

Surgical extrusion is faster but demands an atraumatic approach to minimize damage to cementum and prevent complications such as ankylosis or bone loss [16]. Treatment modality selection depends on patient‐specific factors like esthetic expectations, root anatomy, biologic width, and periodontal health [17]. To achieve this goal, atraumatic extraction systems were developed and marketed under different brand names, which can be used for both regular and atraumatic extraction/extrusion [18]. A previous animal study on the efficacy of an atraumatic extraction system showed that it resulted in lower cementoblast loss compared with the conventional methods and was beneficial for this purpose [19].

Considering all the above, the present case report describes the treatment process and outcome of a maxillary canine tooth, which was a candidate for extrusion by the atraumatic extractor winch.

## 2 Case History/Examination

**FIGURE 1:**
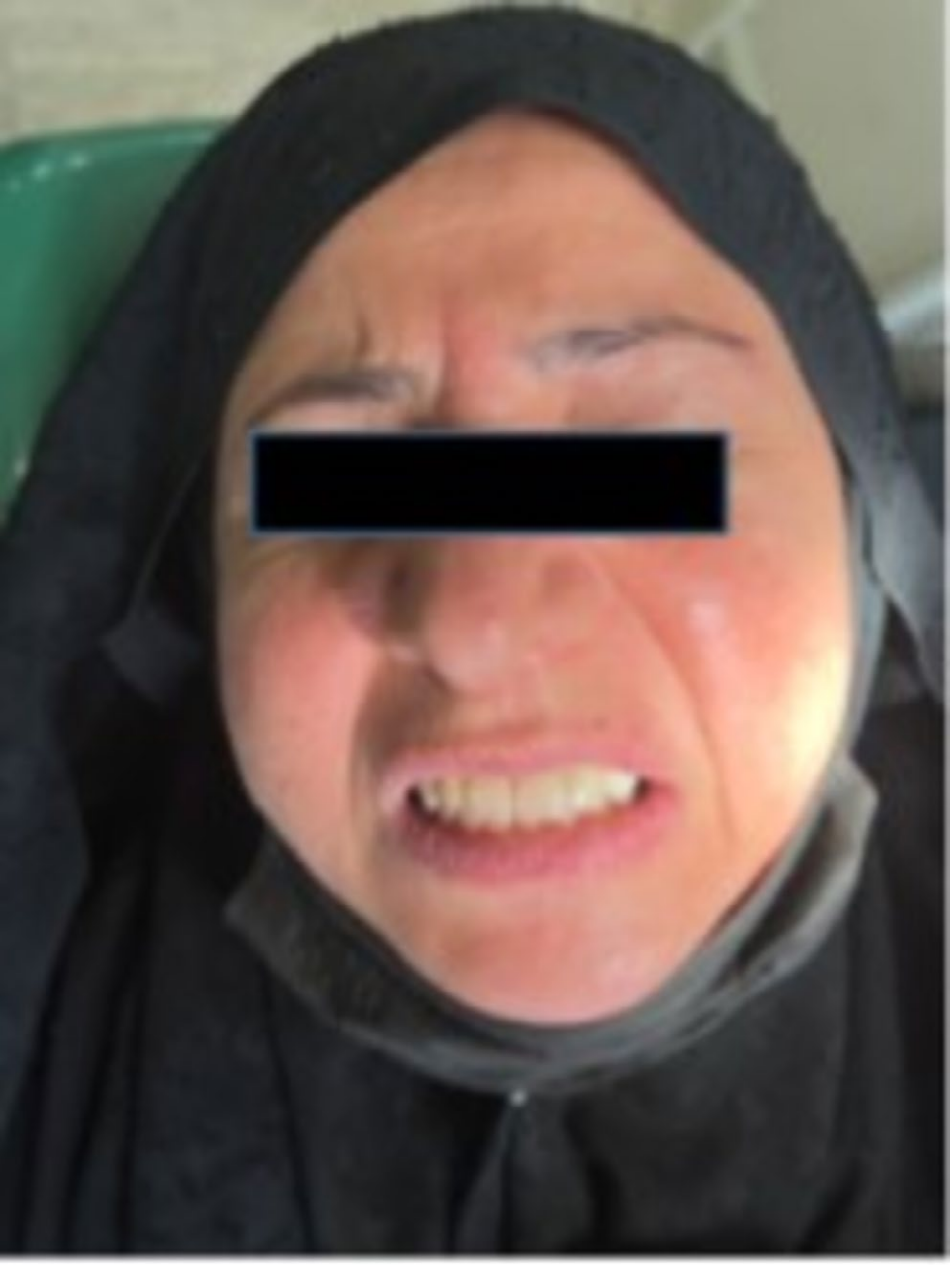
Extraoral clinical view showing no facial asymmetry or swelling.

A 59‐year‐old woman was referred by her dentist to the Endodontics Department of School of Dentistry of Tehran University of Medical Sciences, with the chief complaint of fracture and unesthetic appearance of her left maxillary canine tooth, which had a cervical carious lesion. A review of the patient's medical history revealed that she had controlled hypertension, which was unrelated to her dental condition, and she was classified as ASA II. Extraoral clinical examination revealed no edema or noticeable facial asymmetry (Figure 1).

**FIGURE 2:**
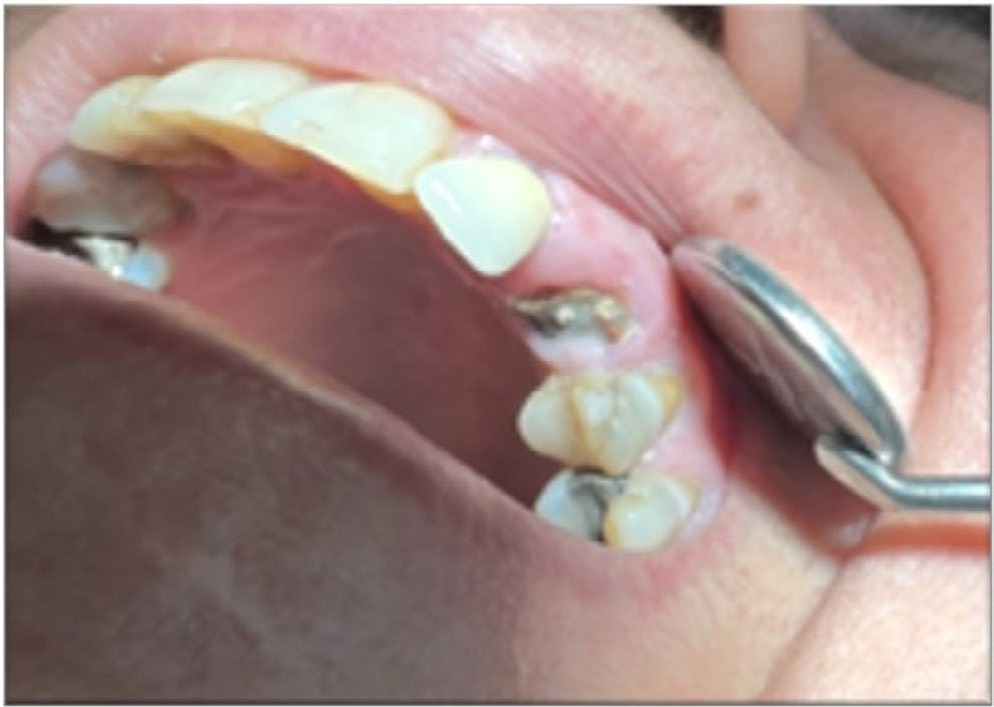
Intraoral view of the left maxillary canine with severe coronal destruction and cervical caries.

**FIGURE 3:**
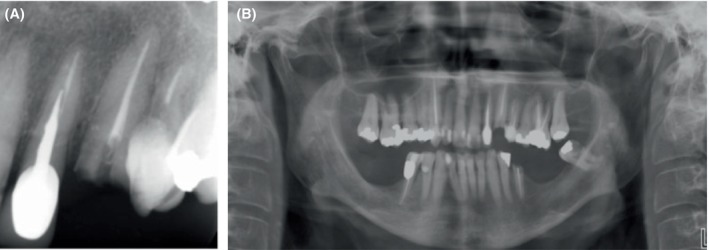
Preoperative radiographs. (A) Periapical radiograph showing an underfilled root canal with no periapical pathology, (B) Panoramic radiograph confirming the absence of periapical lesions.

Intraoral clinical examination revealed a left maxillary canine with a severely destructed crown and poor oral hygiene (Figure 2). Pulp vitality tests of the respective tooth were all negative since the tooth had undergone root canal therapy 4 years earlier. The palpation, percussion, and mobility test results were all within the normal range. The tooth had a probing depth of 4 mm. Periapical and panoramic radiographs revealed a root filling shorter than the apex (under‐filling) with no periapical pathosis (Figure 3A,B).

## 3 Differential Diagnosis, Investigations, and Treatment

Due to the extensive coronal destruction, the sound tooth structure was 3–4 mm apical to the gingival margin and the alveolar crest; therefore, 4 mm of extrusion was required to provide a ferrule and sufficient biologic width for the definitive restoration. The available treatment options included (I) endodontic retreatment and coronal extrusion by the orthodontic or the surgical technique for the definitive restoration, (II) endodontic retreatment and crown lengthening by surgery, and (III) tooth extraction and implant placement. Considering factors such as the patient's age, root structure of the canine tooth, smile line, oral hygiene status, patient's preference for tooth preservation, and giving consent for the procedure, the first option was selected after consultation with a periodontist to ensure that the tooth can be preserved.

Since coronal fracture can compromise the integrity of the tooth structure and cause recontamination of the root canal system, any delay in placement of definitive restoration after root canal therapy can increase the risk of coronal leakage and necessitate endodontic retreatment. Also, considering the possibility of the presence of untreated missed canals in primary endodontic treatment, endodontic retreatment could help in complete elimination of infection and a suitable root canal preparation for a new reliable restoration [20]. Thus, endodontic retreatment was considered prior to coronal reconstruction for this case.

### 3.1 First Treatment Session

**FIGURE 4:**
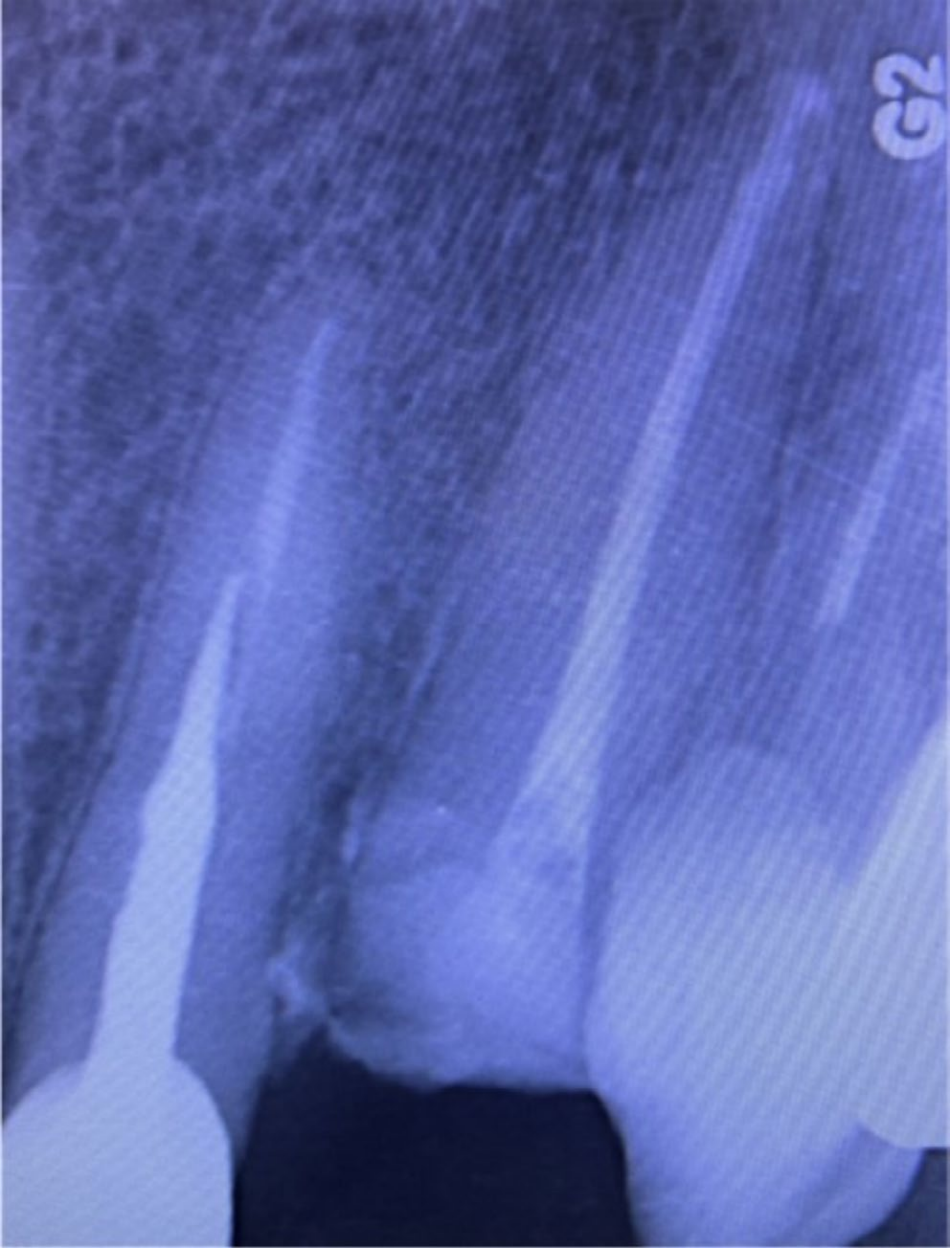
Post‐obturation periapical radiograph after endodontic retreatment using cold lateral compaction.

Considering the under‐filled root canal as detected on the periapical radiograph, absence of optimal coronal seal, and the possibility of root canal contamination, endodontic retreatment was scheduled for the patient. Cleaning and shaping of the canal were performed by the step‐back technique using Denco rotary file system (Denco, Shenzhen, China) in S2, F1, and F2 sizes to the canal length (21 mm). The root canal was subsequently obturated with gutta‐percha (Meta; Meta Biomed, Cheongju, South Korea) by the cold lateral compaction technique (Figure 4).

### 3.2 Second Treatment Session

Winch extractor (Daimotech, South Korea) is a professional tool used for tooth extrusion, especially for avulsed or dislocated teeth. It applies gradual controlled force to the tooth, which is imperative when a tooth needs gentle repositioning or extraction without traumatizing the bone or the periodontal ligament. The winch extractor includes a mechanical system which is attached to the tooth and applies gentle force for tooth extraction from the socket through a clamp or a ring; accordingly, the risk of root fracture and alveolar bone or soft tissue damage is minimized. Controlled tooth extrusion is particularly valuable in orthodontics and endodontics, as precise management of tooth movement can prevent complications, preserve tooth integrity, and minimize damage from uneven or excessive force often seen with traditional extraction methods [21].

**FIGURE 5:**
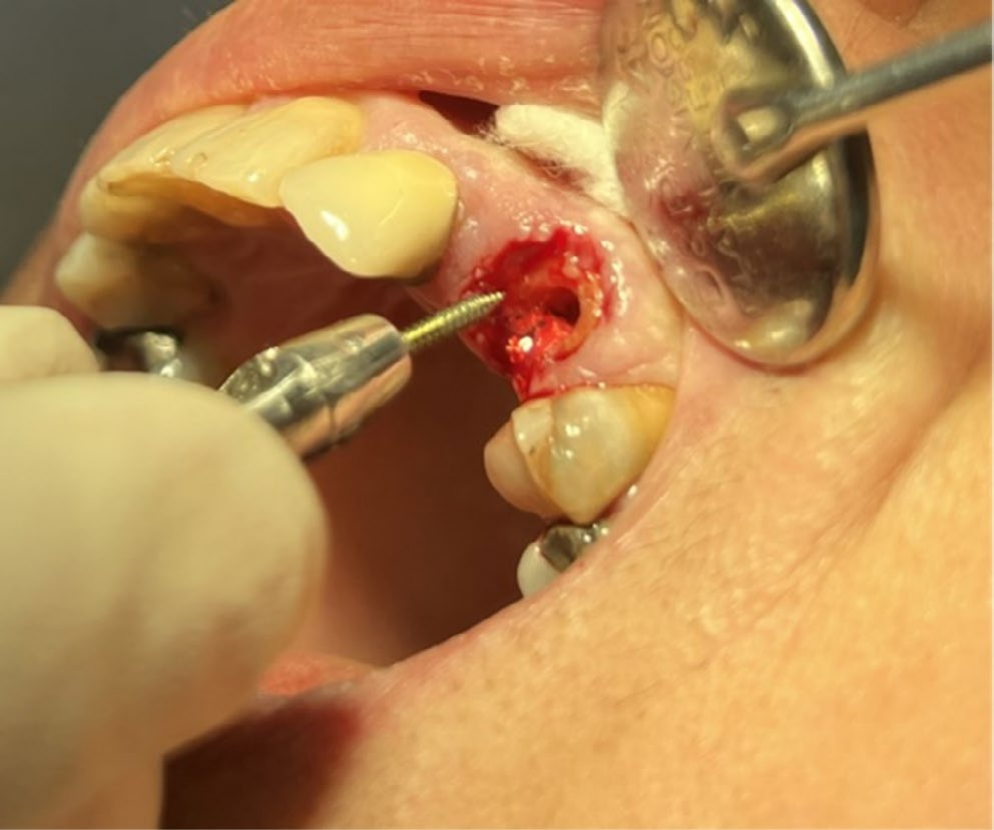
Insertion of the winch extractor screw into the previously prepared and emptied root canal prior to controlled atraumatic extrusion.

**FIGURE 6:**
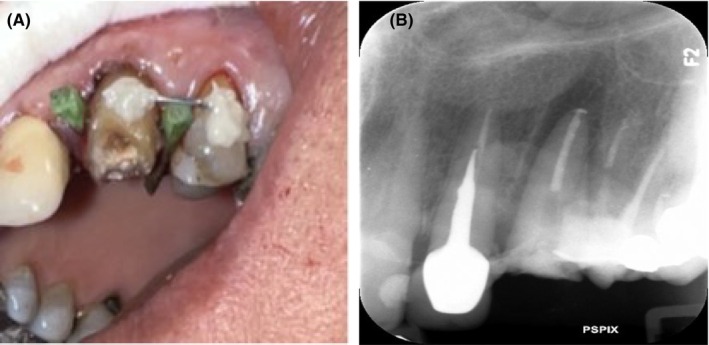
Post operative views: (A) Clinical view of the extruded maxillary canine stabilized with a wire splint to the adjacent tooth; (B) Periapical radiographic view demonstrating coronal repositioning of the tooth relative to its original socket position.

Gutta‐percha was removed from the root canal by 10 mm using a #2 peeso reamer (Figure 5). Next, the respective pin and screw were introduced into the canal by rotational movement almost passively but firmly. The device was then connected to the pin, and controlled root extrusion was performed by 3–4 mm within 2 min, as instructed by the manufacturer (Figure 6A,B). The tooth was rigidly splinted to the adjacent tooth by using a 0.7 wire and Denfill composite resin (Denmat, Lompoc, CA, USA) for 2 weeks. Supporting Information (Movie S1) shows the abovementioned process in a short video.

### 3.3 Third Treatment Session

Two weeks after debonding of the splint, tooth mobility was evaluated and since it was grade I (< 1 mm) according to the Miller's mobility index [22], the tooth was not splinted again. Finally, the patient was referred to a prosthodontist for placement of the definitive restoration (post and core and crown).

## 4 Conclusion and Results

**FIGURE 7:**
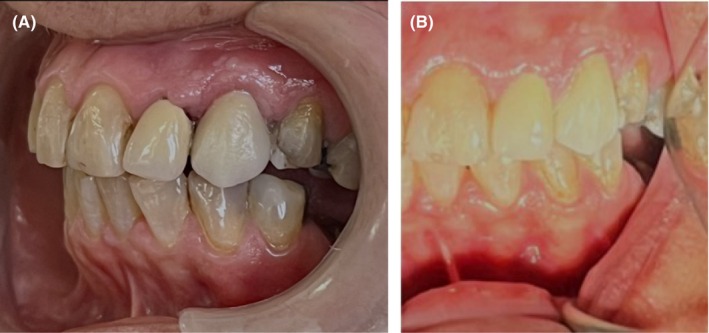
Extraoral clinical photographs of the restored maxillary canine. (A) Oblique view showing proper placement of the crown and harmonious alignment with adjacent teeth. (B) Frontal view demonstrating adequate gingival margins with no signs of inflammation and optimal esthetic integration.

**FIGURE 8:**
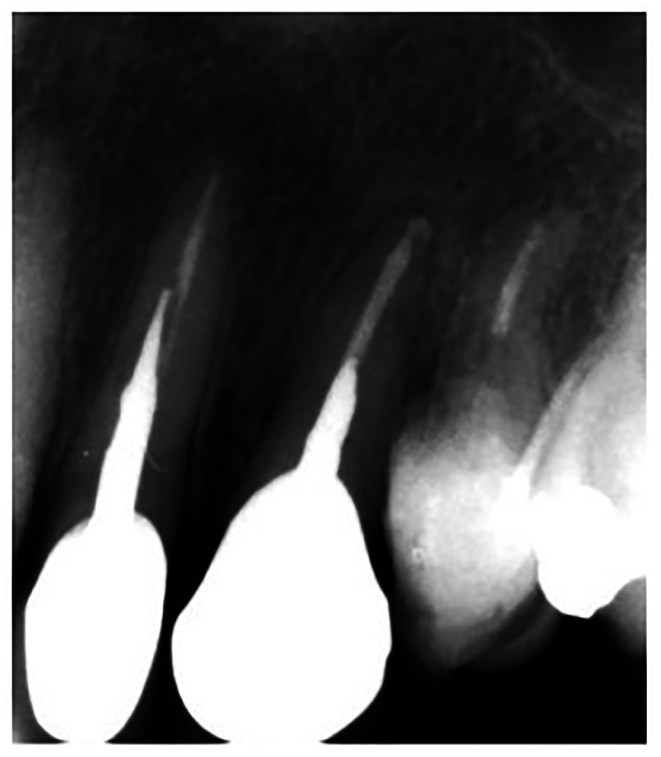
Twelve‐month follow‐up periapical radiograph demonstrating satisfactory bone healing and absence of root resorption around maxillary canine.

The patient could not show up for a follow‐up due to back surgery for 6 months after the treatment. Thus, the first follow‐up examination was performed 12 months after the extrusion treatment. The patient had no complaints, and intraoral examination revealed that the tooth was asymptomatic (Figure 7A,B). The tooth's response to the palpation and percussion tests, and its probing depth were all within the normal range. Also, the tooth was not mobile when tested according to Miller's method. Radiographs showed optimal bone healing around the root, with no evidence of root resorption (Figure 8). Written informed consent was obtained from the patient for publication of this case report and accompanying images.

## 5 Discussion

Teeth with extensive coronal destruction present significant clinical challenges, including difficulty in isolation for adhesive procedures, insufficient residual tooth structure for predictable restoration, and placement of restorative margins deep within the periodontal tissues. Atraumatic extrusion addresses these limitations by repositioning the sound root structure coronally, which allows proper isolation, establishment of a ferrule, and relocation of the restorative margin to a more favorable supragingival position, while preserving periodontal health.

Preservation of natural teeth, particularly in the anterior esthetic zone, is often a more conservative and biologically favorable approach than immediate extraction and dental implant placement. Natural teeth maintain proprioception via the periodontal ligament, which allows for the regulation of occlusal force and adaptation to masticatory demands. In contrast, implants lack this sensory feedback, potentially leading to overloading and long‐term complications in occlusal dynamics. Additionally, the presence of a root in the socket stimulates the surrounding alveolar bone during mastication, preventing resorption. Implant placement, especially in areas with limited buccal bone, often requires additional surgical interventions such as grafting procedures to achieve long‐term success and esthetic harmony [23].

Recent advancements in atraumatic extraction and extrusion systems have provided clinicians with less invasive and more predictable alternatives for managing teeth previously considered non‐restorable. The Benex system (Helmut Zepf Medizintechnik GmbH, Germany) utilizes an intra‐radicular screw and a vertical traction mechanism that minimizes lateral force application. This design effectively reduces damage to the periodontal ligament and surrounding alveolar bone, lowering the risk of root and bone resorption during extrusion [24]. The winch extractor, used in the present case, applies a gradual tensile force via a rotating mechanism. While less technically complex and more accessible, it offers less control than the Benex system and is more operator‐dependent [21].

Despite their benefits, these systems are not without complications. Krug et al. reported that 82.3% of non‐restorable teeth treated with the Benex system were successfully extruded and restored with minimal clinical or radiographic complications [19]. However, surgical extrusion may lead to issues such as non‐progressive root resorption (up to 30%), tooth loss (5%), slight mobility (4.6%), and marginal bone loss (3.7%) [16, 19]. The transient root resorption rate has been estimated at 9.8%, while technical complications such as screw misalignment and root fracture occurred in approximately 9.7% of cases [19].

Procedural errors are not uncommon and are typically related to incorrect screw positioning, inappropriate root anatomy, or excessive force application. Root perforation is a known risk, especially in teeth with calcified canals, where creating a path for screw insertion becomes challenging. Long‐term risks such as dentinal microcrack formation further highlight the need for careful technique and case selection [19, 25]. Moreover, post‐extrusion complications such as temporary root resorption and severe coronal discoloration (up to 54%) may occur, impacting both function and esthetics [26].

Choosing between performing root canal therapy before or after extrusion depends on the clinical scenario. In traumatized teeth, extrusion prior to endodontic treatment can aid in detecting fractures and facilitate rubber dam placement. Conversely, in non‐traumatized teeth—such as the present case—where isolation is possible and retreatment is required, endodontic therapy can be completed before extrusion [27].

Economic and clinical data support the cost‐effectiveness of endodontic retreatment and extrusion over implant placement. Although implant therapy may offer a shorter overall treatment timeline, it typically involves higher costs, more surgical steps, and a greater risk of complications. Studies suggest that short‐term success rates are similar for both approaches, with tooth preservation offering distinct biological and functional advantages [28, 29].

However, atraumatic extrusion is not universally applicable. It is contraindicated in multi‐rooted teeth due to lower success rates (43% vs. 89% for single‐rooted teeth) and increased risk of root fracture and screw misalignment. Severe root caries may also weaken structural integrity, compromising the effectiveness of the procedure. Operator expertise plays a key role in minimizing such risks and optimizing outcomes [25].

In conclusion, atraumatic extrusion using systems like the Benex and winch extractor represents a viable option for salvaging compromised anterior teeth. When combined with proper endodontic management, careful case selection, and adherence to minimally invasive principles, these systems can provide predictable, cost‐effective outcomes with functional and esthetic benefits. Further research, especially on winch‐based techniques, is necessary to establish long‐term efficacy and refine clinical protocols.

### 5.1 Suggestions

Atraumatic extrusion is a gentle technique; the tooth should be luxated and extruded to the desired level with utmost care to minimize trauma to the marginal bone and root surface. Attention to atraumatic principles during extrusion would preserve the PDL health and lead to better results. Also, more extensive studies are recommended on different atraumatic extraction systems to assess the treatment results and possible complications.

In teeth with coronal destruction, endodontic retreatment along with extrusion can serve as a suitable treatment option to preserve natural dentition and decrease the treatment costs. Different atraumatic extraction systems may be efficiently used for the extrusion of severely damaged teeth. They enable faster treatment by preservation of the periodontium and the supporting bone.

## Author Contributions

**Shamsedin Heydari:** data curation, investigation, methodology, validation, writing – original draft. **Mohsen Aminsobhani:** conceptualization, investigation, supervision. **Foozie Zahedi:** investigation, project administration, writing – original draft, writing – review and editing.

## Funding

The authors have nothing to report.

## Ethics Statement

The authors take full responsibility for the integrity and accuracy of the work and for addressing any concerns related to its content. All clinical procedures were conducted in compliance with the ethical guidelines of the relevant institutional and national committees. Written informed consent was obtained from the patient for publication of this case report and its associated clinical images.

## Conflicts of Interest

The authors declare no conflicts of interest.

## Supporting information


**Movie S1:** Demonstrates the controlled root extrusion process using the winch extractor, including step‐by‐step attachment and gradual tooth elevation.

**Data S1:** ccr372016‐sup‐0002‐DataS1.jpg.

## Data Availability

The authors have nothing to report.
