# Supplementary figures and images for: Atraumatic Maxillary Canine Root Repositioning as an Alternative to Orthodontic Forced Eruption: A Case Report
Source: Clin Case Rep. 2026 Feb 10;14(2):e72016. doi: 10.1002/ccr3.72016 (PMC12887680; doi:10.1002/ccr3.72016)

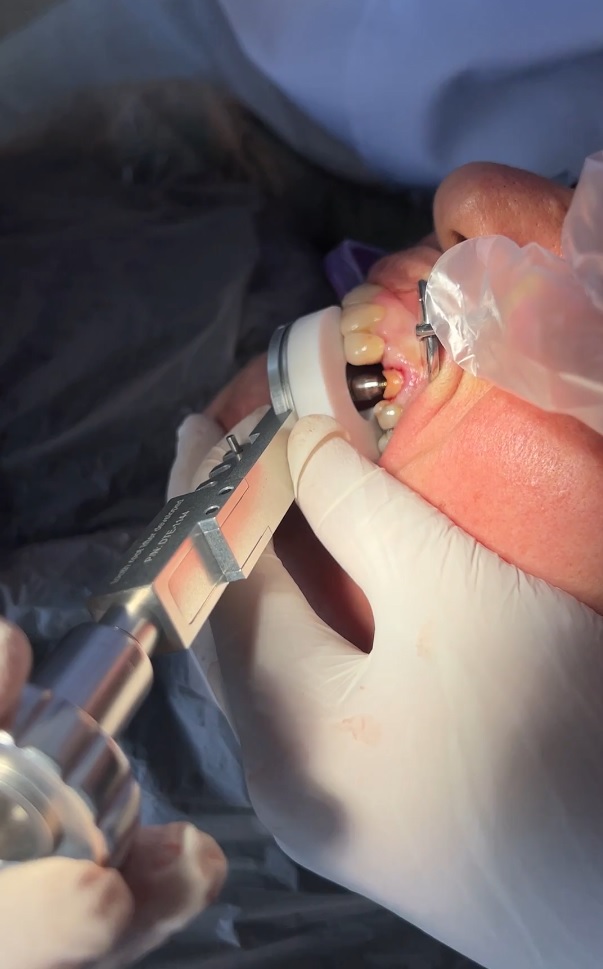

Supplement: Supplementary file 2 — Data S1: ccr372016‐sup‐0002‐DataS1.jpg. [file CCR3-14-e72016-s002.jpg]
